# Non-Lethal Ionizing Radiation Promotes Aging-Like Phenotypic Changes of Human Hematopoietic Stem and Progenitor Cells in Humanized Mice

**DOI:** 10.1371/journal.pone.0132041

**Published:** 2015-07-10

**Authors:** Changshan Wang, Motohiko Oshima, Goro Sashida, Takahisa Tomioka, Nagisa Hasegawa, Makiko Mochizuki-Kashio, Yaeko Nakajima-Takagi, Yoichiro Kusunoki, Seishi Kyoizumi, Kazue Imai, Kei Nakachi, Atsushi Iwama

**Affiliations:** 1 Department of Cellular and Molecular Medicine, Graduate School of Medicine, Chiba University, Chiba 260–8670, Japan; 2 College of Life Sciences, Inner Mongolia University, Hohhot 010021, China; 3 International Research Center for Medical Sciences, Kumamoto University, Kumamoto City 860–0811, Japan; 4 Department of Hematology, Chiba University Hospital, Chiba 260–8670, Japan; 5 Department of Radiobiology/Molecular Epidemiology, Radiation Effects Research Foundation, Hiroshima 732–0815, Japan; B.C. Cancer Agency, CANADA

## Abstract

Precise understanding of radiation effects is critical to develop new modalities for the prevention and treatment of radiation-induced damage. We previously reported that non-lethal doses of X-ray irradiation induce DNA damage in human hematopoietic stem and progenitor cells (HSPCs) reconstituted in NOD/Shi-*scid* IL2rγ*^null^* (NOG) immunodeficient mice and severely compromise their repopulating capacity. In this study, we analyzed in detail the functional changes in human HSPCs in NOG mice following non-lethal radiation. We transplanted cord blood CD34^+^ HSPCs into NOG mice. At 12 weeks post-transplantation, the recipients were irradiated with 0, 0.5, or 1.0 Gy. At 2 weeks post-irradiation, human CD34^+^ HSPCs recovered from the primary recipient mice were transplanted into secondary recipients. CD34^+^ HSPCs from irradiated mice showed severely impaired reconstitution capacity in the secondary recipient mice. Of interest, non-lethal radiation compromised contribution of HSPCs to the peripheral blood cells, particularly to CD19^+^ B lymphocytes, which resulted in myeloid-biased repopulation. Co-culture of limiting numbers of CD34^+^ HSPCs with stromal cells revealed that the frequency of B cell-producing CD34^+^ HSPCs at 2 weeks post-irradiation was reduced more than 10-fold. Furthermore, the key B-cell regulator genes such as *IL-7R* and *EBF1* were downregulated in HSPCs upon 0.5 Gy irradiation. Given that compromised repopulating capacity and myeloid-biased differentiation are representative phenotypes of aged HSCs, our findings indicate that non-lethal ionizing radiation is one of the critical external stresses that promote aging of human HSPCs in the bone marrow niche.

## Introduction

Human hematopoiesis is a critical physiological regenerative process that provisions mature blood cell lineages. All lineages of cells in the blood system are derived from hematopoietic stem cells (HSCs), which are capable of both self-renewal and multi-lineage differentiation. The self-renewal capacity is an essential feature of HSCs, generating at least one daughter cell that has stem-cell properties similar to the parental cell [1], [2]. Stem-cell exhaustion caused by impaired self-renewal capacity of stem cells determines the aging phenotypes of tissues and organs and thus has been recognized as one of the hallmarks of mammalian aging [3]. It also underlies certain degenerative diseases [4]. HSCs show several characteristic phenotypes with age, such as impaired homing and repopulating capacity and biased differentiation toward myeloid lineage at the expense of their lymphoid potential [5–11]. However, aging phenotypes have not been fully evaluated for human HSCs *in vivo*.

Genomic stability is extremely important for the normal physiology of hematopoietic system and for preventing hematopoietic failure. Ionizing radiation that induces DNA damage is a major risk factor for genomic instability. Accumulation of DNA damage and loss of DNA repair capacity are generally associated with apoptosis, cellular senescence, cancer and stem cell abnormalities [12]. Of note, accumulation of DNA damage in HSCs has been invoked to explain an age-related decline in stem-cell function [13–17]. One mechanism through which ionizing radiation affects the human hematopoietic system, especially HSCs, is through inducing DNA damage *in vivo*; however, non-lethal radiation effects on human HSCs have not been well characterized *in vivo* due to lack of suitable animal models. Therefore, effects of ionizing radiation have thus far been evaluated mainly in mice or by irradiating human HSCs *ex vivo* [18]. In order to understand non-lethal radiation effects on human hematopoietic stem and progenitor cells (HSPCs) *in vivo*, we previously analyzed NOD/Shi-*scid* IL-2rγ^null^ (NOG) immunodeficient mice reconstituted with human HSCs and preliminarily evaluated the effects of 0.5 and 1.0 Gy of total-body irradiation (TBI) on human HSCs [19], in which we observed that DNA damage inflicted by ionizing radiation restricts the self-renewal capacity of human HSPCs *in vivo*. This humanized mouse model is useful for the evaluation of radiation effects on human hematopoiesis, including DNA damage, particularly in the dose region where long-term effects on the hematopoietic system as well as other organs have been observed among atomic-bomb survivors [20].

In this study, we further evaluated the functional changes in human HSPCs in NOG mice and the effects of non-lethal radiation in this process. Our findings demonstrate that non-lethal doses of ionizing irradiation profoundly affect the function of HSPCs in humanized mice and promote part of the aging-related phenotypes of human HSPCs in humanized mice, including compromised repopulating capacity and myeloid-biased differentiation at the expense of B lymphopoiesis.

## Materials and Methods

### Ethics Statement

Experiments using mice were performed in accordance with institutional guidelines of the Graduate School of Medicine, Chiba University. This study was approved by the Institutional Review Committees of Chiba University (approval numbers 24–64 and 26–131).

### Cord blood cells and transplantation

Human cord blood (hCB) was kindly provided by the Japanese Red Cross Kanto-Koshinetsu Cord Blood Bank (Tatsumi, Tokyo, Japan). CD34^+^ cells were immunomagnetically enriched using a magnetic-activated cell-sorting CD34 progenitor kit (Miltenyi Biotech, Auburn, CA, USA) and were kept frozen. The purity of hCB CD34^+^ cells was approximately 95%. Frozen stock of the CD34^+^ cells was thawed and plated at 5 x 10^5^ cells/well in a 6-well plate pre-coated with 25 μg/mL fibronectin fragment CH-296 (Takara Shuzo, Otsu, Shiga, Japan) and cultured in a serum-free medium, StemSpan SFEM (Stem Cell Technologies, Vancouver, BC, Canada) in the presence of 100 ng/mL human stem cell factor (SCF) (PeproTech, Rocky Hill, NJ, USA) and 50 ng/mL human thrombopoietin (TPO) (PeproTech) for 16 hours. Subsequently, 5 x 10^4^ CD34^+^ cells were injected intravenously into 8-week-old NOG mice which had been irradiated with 2.0 Gy of X-rays 2 hours before. At 2 weeks post-irradiation, human CD34^+^ HSPCs were purified from the bone marrow (BM) of primary recipient mice and 2 x 10^6^ CD34^+^ HSPCs were transplanted into secondary NOG recipients which had been irradiated with 2.0 Gy of X-rays 2 hours before. NOG immunodeficient mice [21] were supplied by the Central Institute for Experimental Animals (Kawasaki, Kanagawa, Japan) and maintained in the Animal Research Facility of the Graduate School of Medicine, Chiba University, in accordance with institutional guidelines.

### Irradiation

Mice were irradiated on a rotating platform of the Hitachi MBR1520R X-ray machine (250 kV; 15 mA; dose-rate: 1.5 Gy/min; 0.5 mm aluminum and 0.1 mm copper filters) (Hitachi, Tokyo, Japan). Irradiation doses were verified by performing dosimetry using TLD100 dosimeters provided by the University of Wisconsin-Madison Radiation Calibration Laboratory (Madison, WI, USA). For 4 weeks after irradiation, mice were fed with drinking water containing 0.17 mg/ml enrofloxacin (Bayer, Shawnee Mission, KS, USA).

### Flow cytometry

Human cells in the BM and PB of recipient mice were stained with Lineage mixture (biotinylated anti-human CD14, CD3, CD56, CD19 and CD235a), PE-Cy7-conjugated anti-human CD34, APC-conjugated anti-human CD45, PE-conjugated anti-human CD38, biotinylated anti-human CD7, biotinylated anti-human CD10, PE-conjugated anti-human CD62L, APC-Cy7-conjugated anti-human CD3, PE-conjugated anti-human CD11b, or PE-Cy7-conjugated anti-human CD19 (BD Biosciences, San Jose, CA, USA). They were then analyzed on a FACS Canto II or sorted on a FACS Aria II (BD Biosciences).

### Colony assay and differentiation of B cells in culture

Human CD34^+^ cells were plated in Methocult GF H4435 methylcellulose medium containing 50 ng/mL human SCF, 10 ng/mL human granulocyte-macrophage colony-stimulating factor (GM-CSF), 10 ng/mL human IL-3, and 3 U/mL human EPO (StemCell Technologies). After 12 to 14 days of culture, the colonies were counted under a microscope. For the detection of B cells, sorted cells were cultured for 4 weeks with TSt-4 stromal cells [22] in 48-well plates. The frequencies of B cell commitment were determined by limiting dilution assays and calculated with L-Calc software (STEMCELL Technologies, Vancouver, BC, Canada). All culture experiments were performed with RPMI 1640 medium supplemented with 10% fetal calf serum, 2 mM L-glutamine, 100 U/ml penicillin, 100 μg/ml streptomycin, 50 μM 2-β-mercaptoethanol, 1 mM sodium pyruvate, 0.1 mM non-essential amino acid solution (Invitrogen, Carlsbad, CA, USA), 20 ng/ml stem cell factor (SCF), 20 ng/ml Flt3 ligand (Flt3L), and 20 ng/ml IL-7 (Peprotech).

### Quantitative real-time PCR analysis

Total RNA was isolated using TRIZOL LS solution (Invitrogen) and reverse transcribed by the ThermoScript RT-PCR system (Invitrogen) with an oligo-dT primer. Real-time quantitative PCR was performed with an ABI prism 7300 Thermal Cycler (Applied Biosystems, Foster City, CA) using FastStart Universal Probe Master (Roche Diagnostics, Branchburg, NJ, USA). *GAPDH* expression was used to calculate relative expression levels. Probe numbers and primer sequences were: Probe #65, 5’-AGGTGGGTAGAGGGTCTGC-3’ and 5’-TCCAATTCCCCTGCAAACT-3’ for *p16* ^*INK4A*^; probe #66, 5’-CACCGCTTTTTCCGAGTG-3’ and CCCCATCGATCCCAATAA-3’ for *HOXA9*, probe 82, 5’-GAGAGGGAAGAGGGGGTGT-37 and 5’-CGCACACAGCTCATACCAAC for *MEIS1*, probe #9, 5’-AGGCACTTTACCTCCACGAG-3’ and GCTTTTGAGGACCCAGATGT-3’ for *IL-7R*, probe #25, 5’-AGGAGCTGCTGCAGTTGG-3’ and 5’-GCTGCCAACTCCCCCTAT-3’ for *EBF1*, probe #83, 5’-CGGCAGCGCTATAATAGTAGG-3’ and 5’-GGAGTGAGTTTTCCGGGAGT-3’ for *PAX5* and probe #25, 5’-CTGACTTCAACAGCGACACC-3’ and 5’-TAGCCAAATTCGTTGTCATACC-3’ for *GAPDH*.

### Statistical analysis

P-values for trend were calculated by nonparametric Jonckheere-Terpstra test. The frequencies of B cell-producing cells in non-irradiated and irradiated cells were compared by the χ2-test for a 2 x 2 contingency table. The t-test was used for comparison of mean values. Statistical analysis was performed using SPSS Statics (version 20, IBM Corp., Chicago, IL, USA).

## Results

### Experimental design to investigate radiation effects on human HSPCs *in vivo*

**Fig 1:**
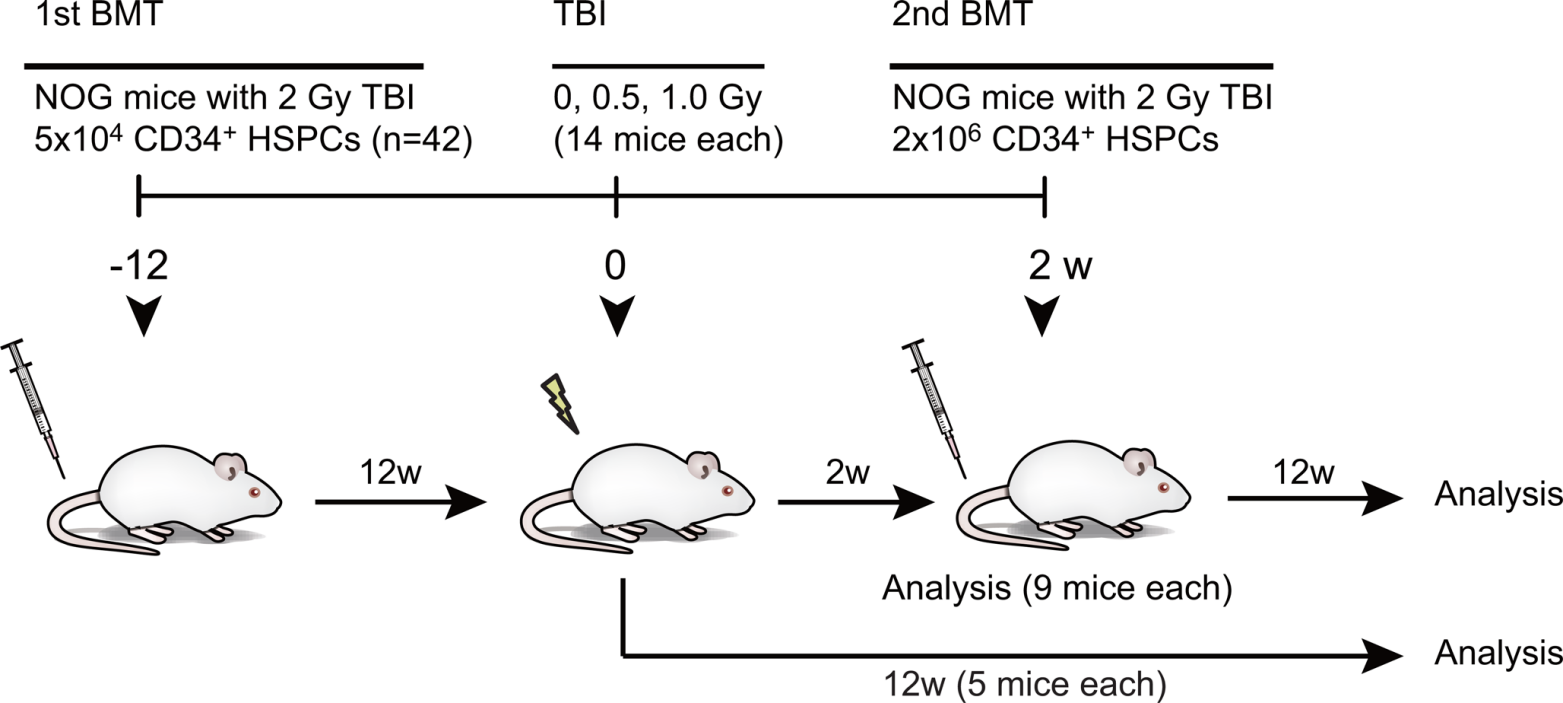
Experimental design to detect the non-lethal radiation effects on human HSPCs *in vivo*. Schema of experimental strategy. Cord blood CD34^+^ HSPCs (5 x 10^4^) were transplanted via tail veins into 8-week-old NOG mice irradiated with 2.0 Gy (n = 42). In the twelfth week after transplantation, the mice were randomly grouped into three categories and irradiated with 0, 0.5 or 1.0 Gy for each group (14 mice each). At 2 weeks post-irradiation, part of the mice (9 mice each) were sacrificed to evaluate early effects of irradiation. At the same time, human CD34^+^ HSPCs were purified from the primary recipient mice (9 mice were pooled), and 2 x 10^6^ CD34^+^ HSPCs were transplanted into the secondary NOG recipients. At 12 weeks post-secondary transplantation, mice were sacrificed to evaluate long-lasting effects of irradiation. The remaining primary recipient mice (5 mice each) were similarly analyzed at 12 weeks post-irradiation to evaluate long-lasting effects of irradiation.

We previously reported that 0.5 or 1.0 Gy of X-ray TBI induced prolonged DNA damage in human HSPCs reconstituted in NOG mice and severely compromised their repopulating capacity [19]. To more deeply understand the effects of low-dose irradiation on human HSPCs, we analyzed the functional changes in human HSPCs in NOG mice following non-lethal irradiation in detail (Fig 1). We prepared a new cohort of humanized mice for this study. We transplanted cord blood CD34^+^ HSPC into NOG mice irradiated with 2.0 Gy. At 12 weeks after the primary transplantation, the NOG mice showed considerably high chimerism, i.e., the median percentage of human CD45^+^ hematopoietic cells in the peripheral blood (PB) was 57.2% (range: 32.0 to 80.6%, n = 42). The mice were randomly grouped into three categories and irradiated with 0, 0.5 or 1.0 Gy for each group (14 mice each), and the radiation effects on human HSPC *in vivo* were assessed (Fig 1).

### Early effects of radiation on human HSPCs in humanized mice

**Fig 2:**
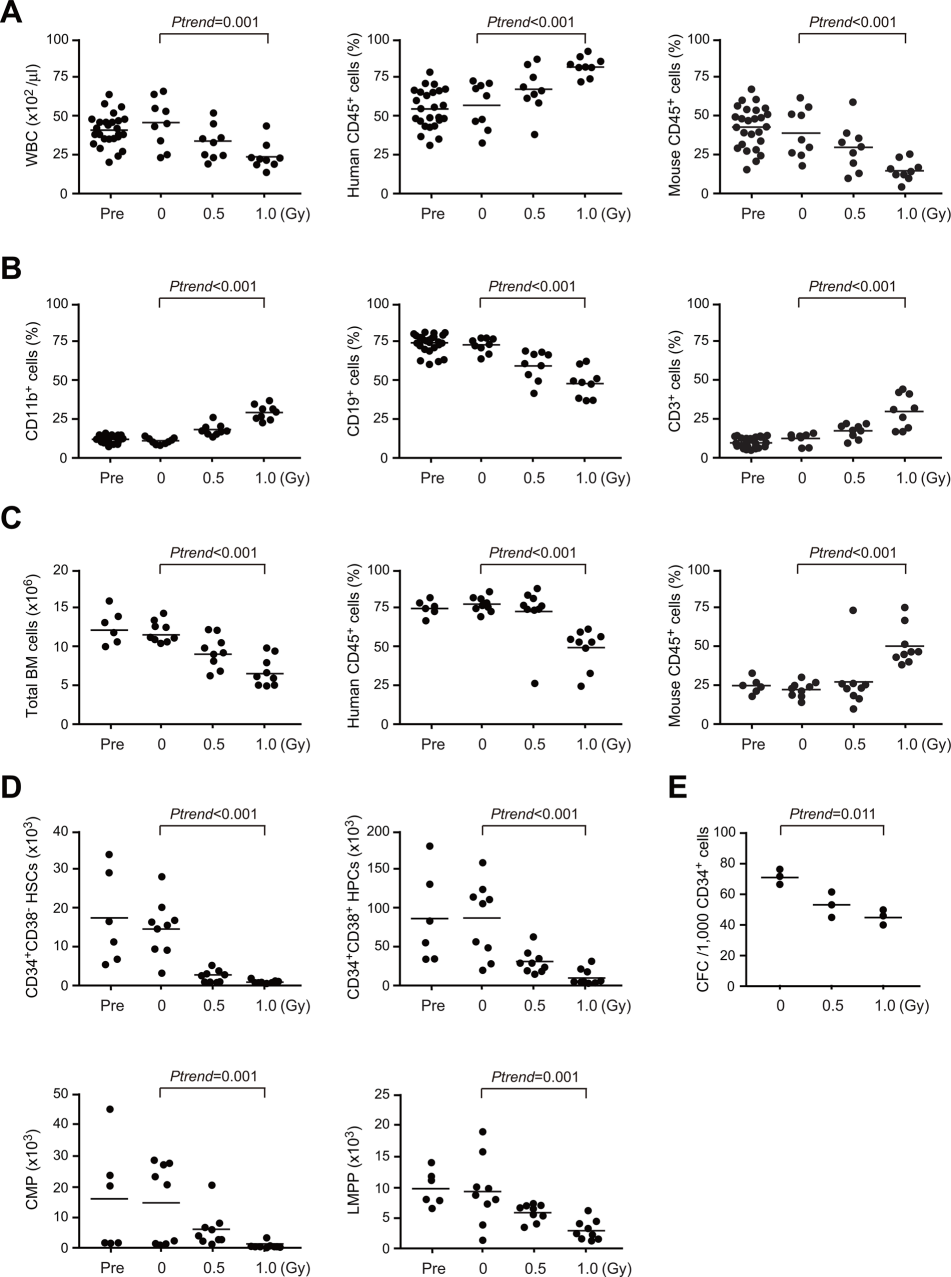
Early effects of irradiation on human HSPCs in humanized mice. (A) Changes in chimerism of human hematopoietic cells in the PB after irradiation. At 12 weeks post-irradiation, the mice were randomly grouped into three categories and irradiated with 0, 0.5 or 1.0 Gy for each group. At 2 weeks post-irradiation, mice were sacrificed to evaluate early effects of irradiation. White blood cell (WBC) counts in the PB (left panel) and chimerism of CD45^+^ human (middle panel) and mouse (right panel) hematopoietic cells in the PB are presented as scatter diagrams with median values (bars) (n = 9 each). PB data of NOG mice just before the irradiation (Pre) are presented as controls (n = 27). (B) The percentages of CD11b^+^ myeloid cells, CD19^+^ B cells, and CD3^+^ T cells in CD45^+^ human hematopoietic cells in the PB of NOG mice just before the irradiation (Pre, n = 27) and at 2 weeks after irradiation (0–1.0 Gy, n = 9 each). (C) Total BM cell numbers (left panel) and chimerism of CD45^+^ human (middle) and mouse (right) hematopoietic cells in the BM just before the irradiation (Pre, n = 6) and at 2 weeks after irradiation (0–1.0 Gy, n = 9 each). (D) Absolute numbers of CD34^+^CD38^-^ HSCs, CD34^+^CD38^+^ HPCs, CMPs and LMPPs in the BM (bilateral femurs and tibiae) just before the irradiation (Pre, n = 6) and 2 weeks after irradiation (0–1.0 Gy, n = 9 each). (E) Numbers of colony-forming cells included in 1,000 CD34^+^ human HSPCs recovered at 2 weeks after irradiation (0–1.0 Gy, n = 3 each). Bars in scatter diagrams indicate median values. *P*-values for trend were calculated by nonparametric Jonckheere-Terpstra test.

**Fig 3:**
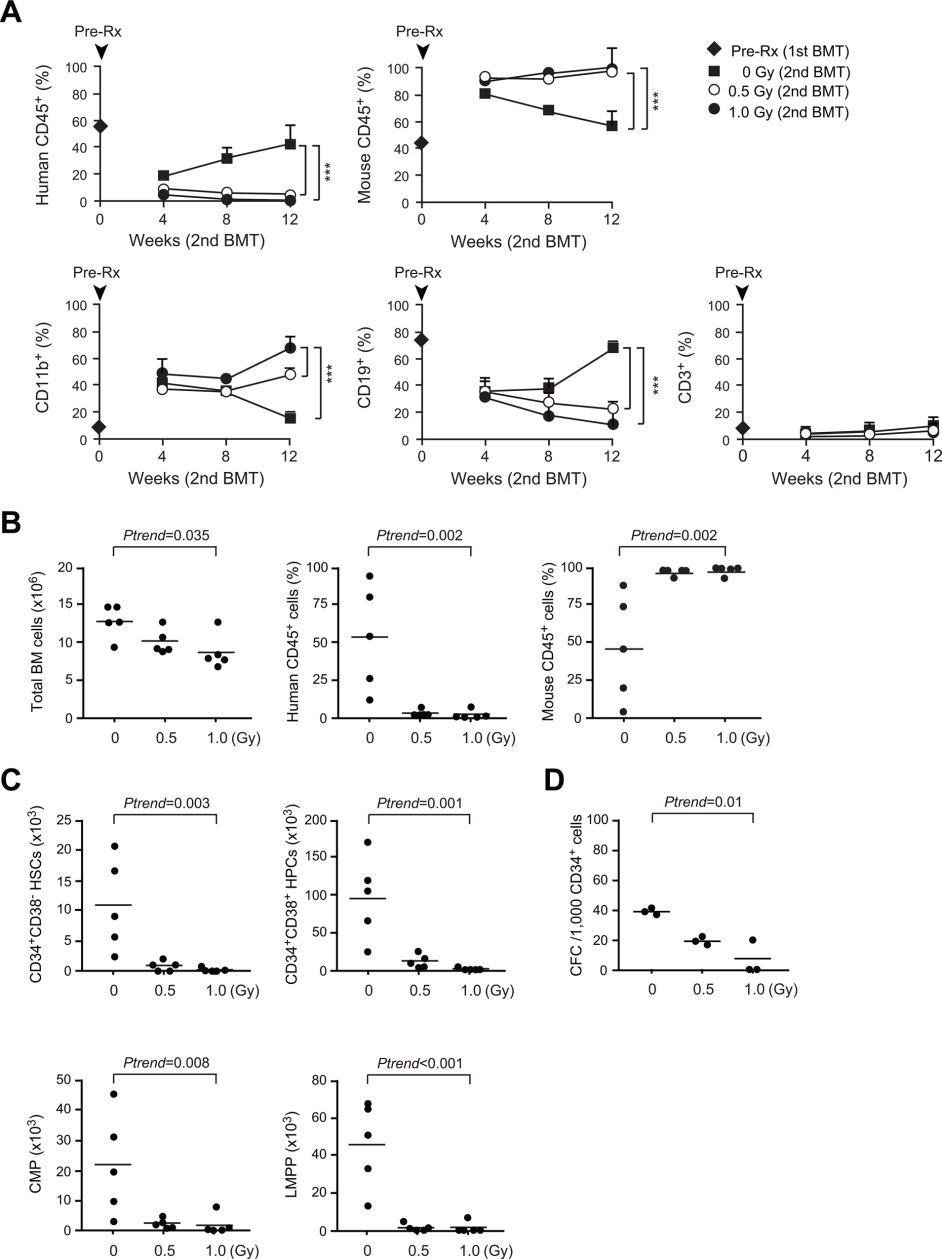
Repopulation of hematopoiesis by irradiated human HSPCs in secondary recipients. (A) Changes in chimerism of human hematopoietic cells in the PB of secondary recipients. At 2 weeks post-irradiation, CD34^+^ human HSPCs were purified from the primary recipient mice, and 2 x 10^6^ CD34^+^ HSPCs were transplanted into secondary NOG recipients. Chimerism of CD45^+^ human and mouse hematopoietic cells in the PB are shown in upper panels. The percentages of CD11b^+^ myeloid cells, CD19^+^ B cells, and CD3^+^ T cells in CD45^+^ human hematopoietic cells in the PB are shown in lower panels. PB data of NOG mice just before the irradiation (Pre, n = 27) are also indicated at week 0. Data are presented as mean ± S.D. (0 Gy, n = 5; 0.5 Gy, n = 5; 1.0 Gy, n = 5). (B) Total BM cell numbers and chimerism of CD45^+^ human and mouse hematopoietic cells at 12 weeks after secondary transplantation. (C) Absolute numbers of CD34^+^CD38^-^ HSCs, CD34^+^CD38^+^ HPCs, CMPs and LMPPs in the BM (bilateral femurs and tibiae) (0 Gy, n = 5; 0.5 Gy, n = 5; 1.0 Gy, n = 5). (D) Numbers of colony-forming cells included in 1,000 CD34^+^ human HSPCs recovered from the BM 12 weeks after secondary transplantation (0–1.0 Gy, n = 3 each). The data in Fig 3B-D are presented as scatter diagrams with median values (bars). *P*-values for trend were calculated by nonparametric Jonckheere-Terpstra test.

**Fig 4:**
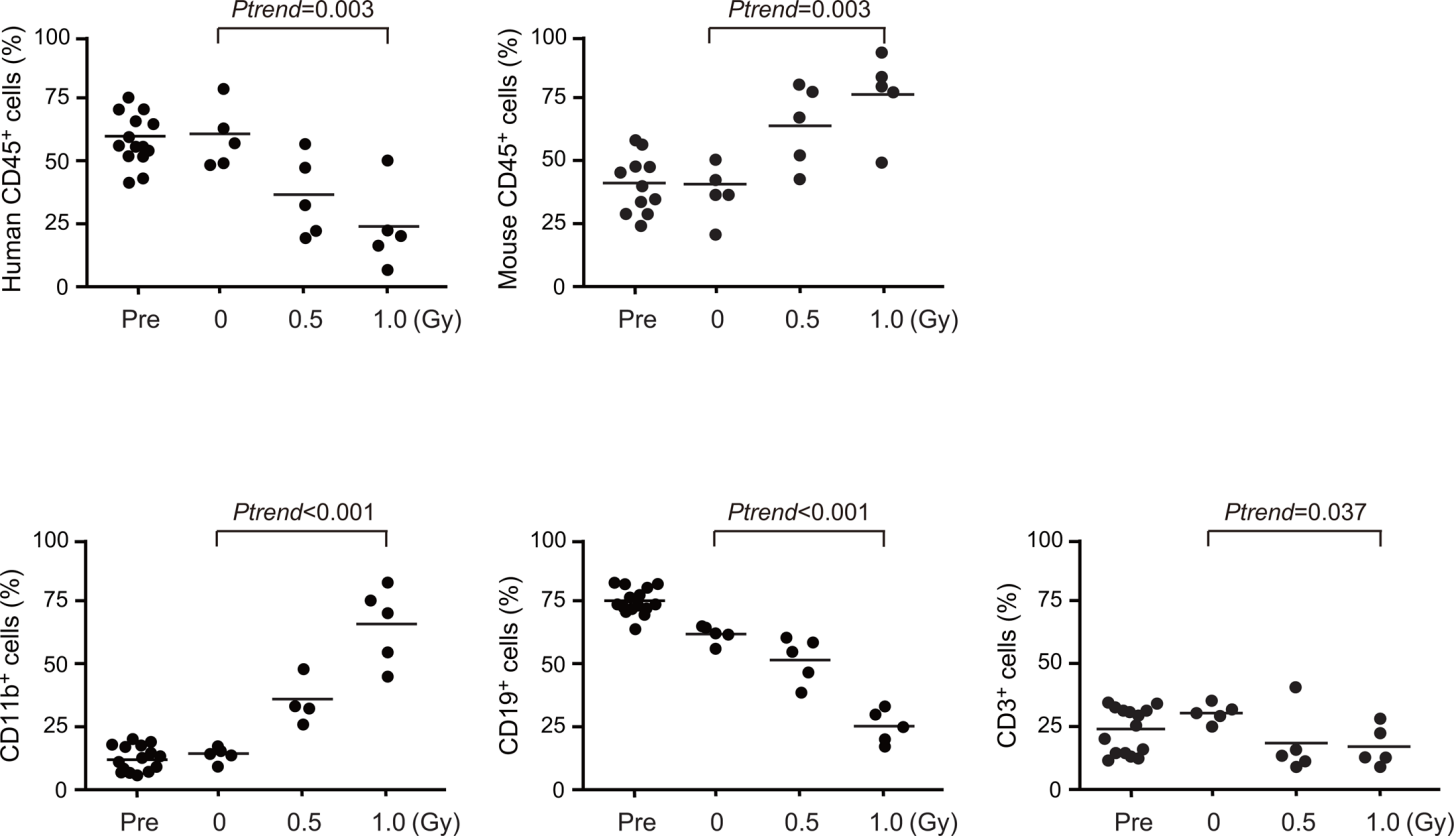
Long-term radiation effects on human HSPCs in primary recipient mice. Changes in chimerism of human hematopoietic cells in the PB of primary recipients at 12 weeks post-irradiation. Chimerism of CD45^**+**^ human and mouse hematopoietic cells in the PB are shown in upper panels. The percentages of CD11b^**+**^ myeloid cells, CD19^**+**^ B cells, and CD3^**+**^ T cells in CD45^**+**^ human hematopoietic cells in the PB are shown in lower panels. PB data of NOG mice just before the irradiation (Pre) are also indicated. Data are presented as scattering diagrams with medium values (bars) (Pre, n = 15; 0 Gy, n = 5; 0.5 Gy, n = 5; 1.0 Gy, n = 5). *P*-values for trend were calculated by nonparametric Jonckheere-Terpstra test. The t-test was used for comparison of mean values.

At 2 weeks post-irradiation, mice were sacrificed to evaluate early effects of radiation (9 mice each). In contrast to NOD/Rag2^*null*^ IL2rγ^*null*^ mice, hematopoietic cells of NOG mice are susceptible to irradiation due to the *scid*/*scid* background [23]. As expected, chimerism of mouse CD45^+^ hematopoietic cells declined in a radiation dose-dependent manner in the PB while that of human hematopoietic cells increased (Fig 2A). Among human cells in the PB, chimerism of B cells significantly decreased in a radiation dose-dependent manner while that of myeloid and T cells increased (Fig 2B). Of interest, chimerism of human CD45^+^ hematopoietic cells declined in a radiation dose-dependent manner in the BM while that of mouse hematopoietic cells increased probably because of inappropriate support of human HSPCs by mouse BM niche cells (Fig 2C). As a consequence, human cells were gradually outcompeted by mouse cells in the PB afterwards as we have reported previously (Figs 3 and 4) [19]. In the BM, radiation dose-dependent decreases in both proportion (data not shown) and absolute numbers were commonly observed in human CD34^+^CD38^-^ HSCs, CD34^+^CD38^+^ HPCs, CD34^+^CD38^+^CD45RA^-^CD10^-^CD7^-^Lineage marker^-^ common myeloid progenitors (CMPs) and CD34^+^CD38^+^CD45RA^+^CD62L^hi^Lineage marker^-^ lymphoid-primed multipotent progenitors (LMPPs) (Fig 2D). In addition, CD34^+^ HSPCs recovered from recipient mice at 2 weeks post-irradiation gave rise to fewer colonies compared with non-irradiated HSPCs (0 Gy vs. 0.5 Gy, *P* = 0.015 and 0 Gy vs. 1.0 Gy *P* = 0.003 by Dunnett t-test) (Fig 2E).

### Repopulation of hematopoiesis by irradiated human HSPCs in secondary recipients

To evaluate the effects of non-lethal radiation on HSPCs repopulated in NOG mice, we then purified CD34^+^ HSPCs from the primary recipient mice at 2 weeks post-irradiation (9 mice were pooled) and transplanted 2 x 10^6^ CD34^+^ HSPCs into secondary NOG recipients. At 12 weeks post-secondary transplantation, we analyzed the contribution of human HSPCs to hematopoiesis (Fig 3). CD34^+^ HSPCs from irradiated mice showed severely impaired reconstitution capacity in the secondary recipient mice. Chimerism of human CD45^+^ hematopoietic cells in both the PB and BM declined in a radiation dose-dependent manner while that of mouse CD45^+^ hematopoietic cells increased (Fig 3A and 3B). Repopulation of human CD34^+^CD38^-^ HSCs, CD34^+^CD38^+^ HPCs, CMPs and LMPPs in the BM was compromised in both proportion (data not shown) and absolute numbers in a radiation dose-dependent manner (Fig 3C). Of interest, non-lethal irradiation impaired contribution of human hematopoietic cells to the PB cells, particularly CD19^+^ B lymphocytes, which indicates the myeloid-biased repopulation of human hematopoietic cells (Fig 3A). CD34^+^ HSPCs from the secondary recipient mice again gave rise to fewer colonies in a manner dependent on radiation doses to which the cells had been exposed in the primary recipient mice (0 Gy vs. 0.5 Gy, *P* = 0.019 and 0 Gy vs. 1.0 Gy *P* = 0.002 by Dunnett t-test) (Fig 3D). Myeloid-biased hematopoiesis at the expense of B lymphopoiesis was also observed at 12 weeks post-irradiation in primary recipients (5 mice each) (Fig 4). Because impaired reconstituting capacity, myeloid-biased differentiation and inefficient production of B cells are hallmarks of aged HSCs [7], [10], [24], these data indicate that non-lethal doses of radiation accelerate part of the aging phenotypes of human HSPCs in humanized mice.

**Fig 5:**
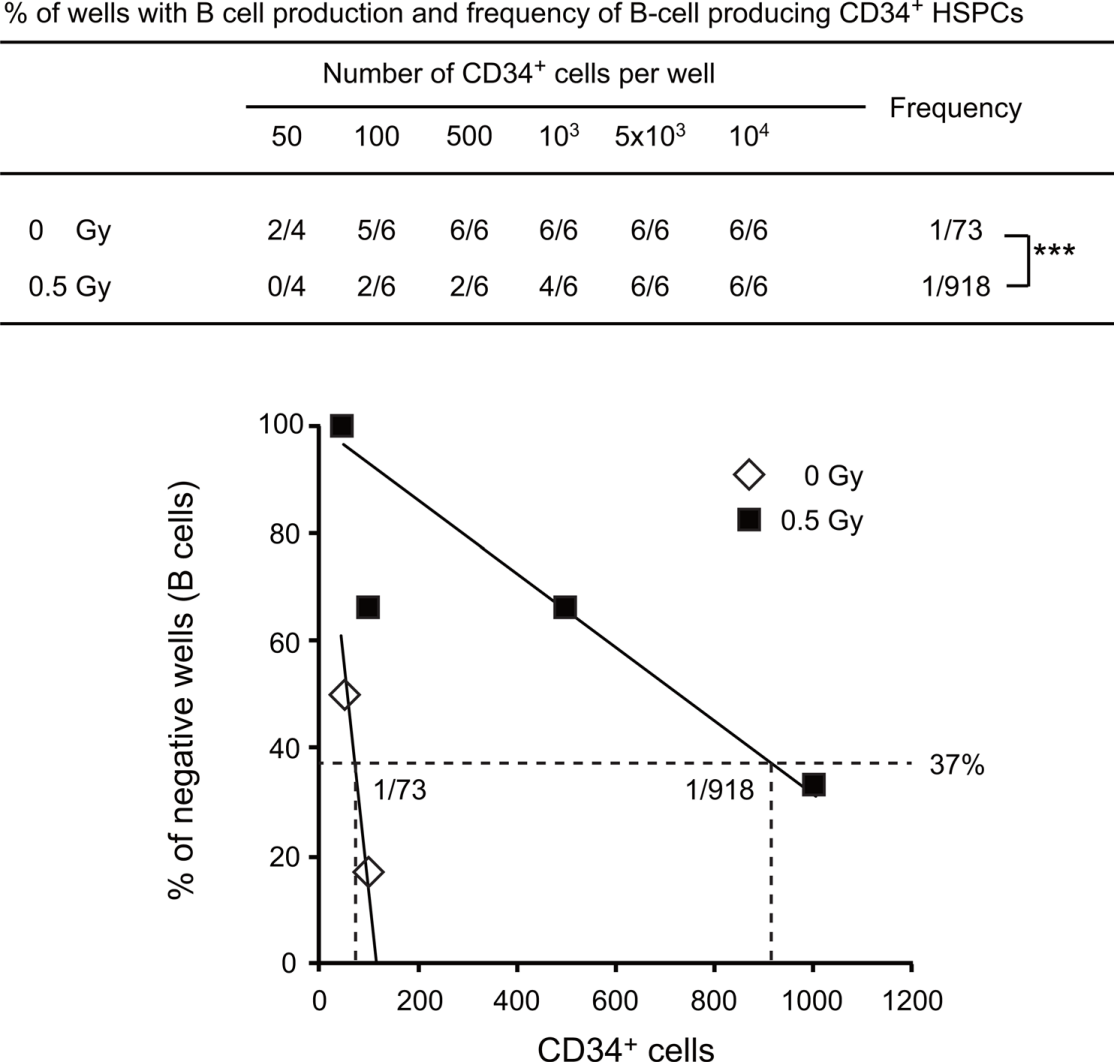
*In vitro* differentiation of B cells from human HSPCs irradiated in humanized mice. Frequency of B cell-producing CD34^**+**^ HSPCs irradiated in humanized mice. Human CD34^**+**^ HSPCs were recovered from the primary recipients at 2 weeks post-irradiation. Graded numbers of purified CD34^**+**^ HSPCs (50, 10^**2**^, 5 x 10^**2**^, 10^**3**^, 5 x 10^**3**^, 10^**4**^ cells) were co-cultured with TSt-4 stromal cells in the presence of SCF, IL-7 and Flt3L (10 ng /ml each) for 4 weeks. Generation of CD19^**+**^ B cells was determined by flow cytometric analysis. The frequency of B–cell producing HSPCs was calculated using L-Calc software. The % of wells with B cell production and frequency of B-cell producing CD34^**+**^ HSPCs are summarized in the table and each data is plotted in the bottom panel. *** *P* < **0.001.** The frequencies in non-irradiated and irradiated cells were compared by the **χ2**-test for a 2 x 2 contingency table.

Little is known about the effects of non-lethal radiation on B-cell production from HSPCs. To understand how non-lethal irradiation impairs B lymphopoiesis, we purified CD34^+^ HSPCs at 2 weeks post-irradiation and co-cultured graded numbers of purified CD34^+^ HSPCs (50, 10^2^, 5x10^2^, 10^3^, 5x10^3^, 10^4^ cells) with TSt-4 stromal cells in the presence of SCF, IL-7 and Flt3L (10 ng /ml each) for 4 weeks and analyzed the differentiation of CD19^+^ B cells. The frequency of B-cell-producing cells was 1/73 in control CD34^+^ HSPCs from the secondary recipients repopulated with non-irradiated CD34^+^ HSPCs. In contrast, the frequency of B cell-producing cells was 1/918 in CD34^+^ HSPCs from the secondary recipients repopulated with irradiated CD34^+^ HSPCs at a dose of 0.5 Gy in primary recipient mice. This frequency of irradiated CD34^+^ HSPCs was 12.5-fold lower than that of non-irradiated cells (Fig 5). A significant reduction in B cell-producing capacity of CD34^+^ HSPCs indicates that radiation damage affects the B cell commitment of CD34^+^ HSPCs.

The molecular mechanisms underlying myeloid-biased differentiation of aged HSPCs remain controversial [6], [10]. However, reduced expression of *EBF1* and *PAX5*, key B-cell regulator genes, has been implicated in this process [25].

**Fig 6:**
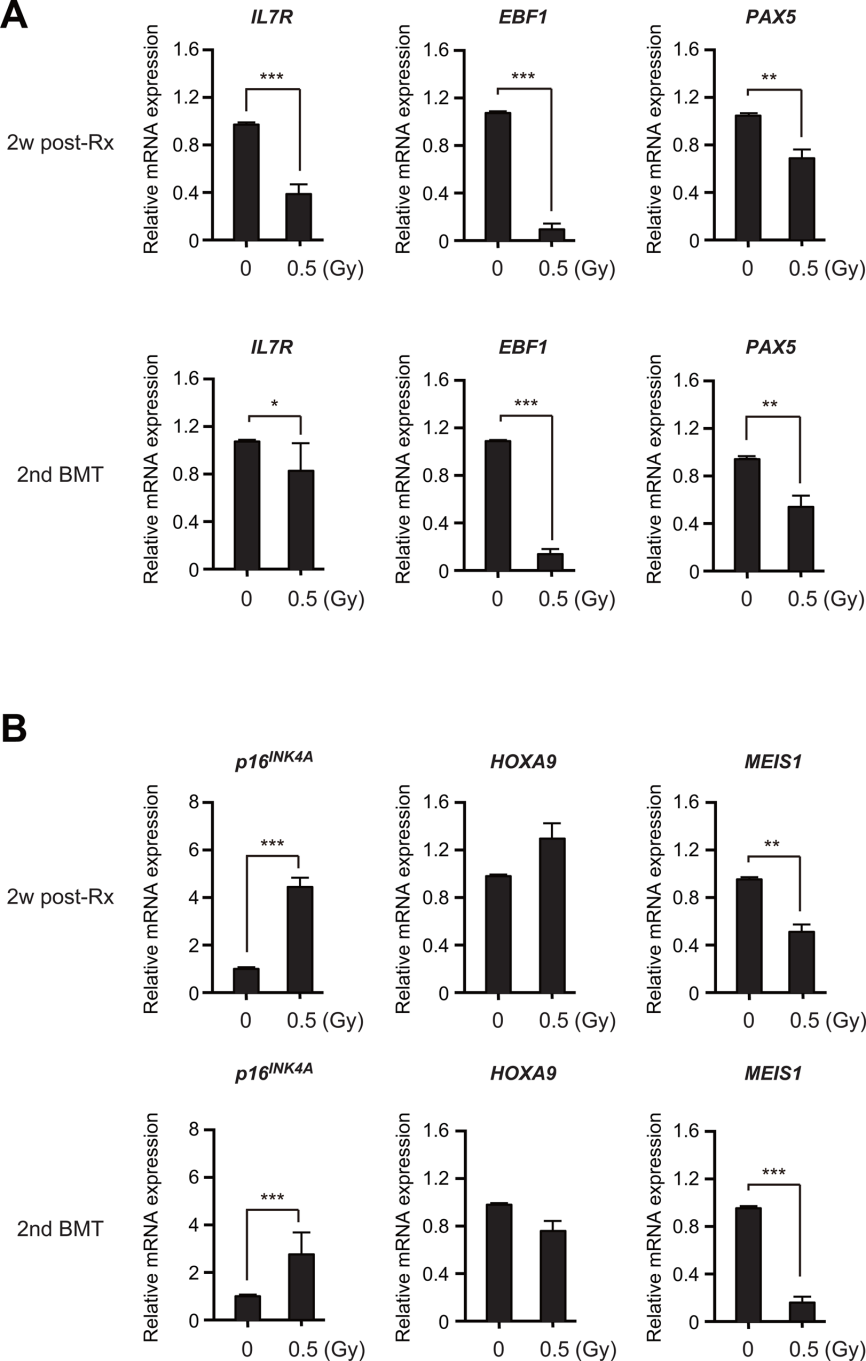
Impaired activation of B cell regulator genes in irradiated human HSPCs. (A) Quantitative RT-PCR analysis of *IL7R*, *EBF1* and *PAX5* in CD34^**+**^ human HSPCs from humanized mice at 2 weeks post-irradiation and at 12 weeks post-secondary transplantation (n = 3 each). (B) Quantitative RT-PCR analysis of *p16INKA*, *HOXA9* and *MEIS1* in CD34^**+**^ human HSPCs from humanized mice at 2 weeks post-irradiation and at 12 weeks post-secondary transplantation (n = 3 each). Relative expression levels of each gene mRNA were adjusted in reference to *GAPDH* mRNA expression levels. Data are shown as the mean e adjust *P* < 0.05, **P < 0.01 and ***P < 0.001. The t-test was used for comparison of mean values.

To obtain an insight into the myeloid-biased differentiation of irradiated HSPCs, we compared the expression levels of each key B-cell regulator gene in CD34^+^ HSPCs from humanized mice at 2 weeks post-irradiation and those at 12 weeks post-secondary transplantation. Of note, expression levels of *IL-7R*, *EBF1* and *PAX5* encoding IL-7 receptor α and the EBF1 and PAX5 master transcription factors, respectively [26], [27] were significantly downregulated upon 0.5 Gy of irradiation (Fig 6A). This trend was conserved in the HSPCs recovered from the primary recipients at 2 weeks post-irradiation and the secondary recipients at 12 weeks post-transplantation (Fig 6A). In addition, the expression of *cyclin-dependent kinase inhibitor 2A* (*CDKN2A*) or *p16* ^*INK4A*^, a hallmark of aging of HSCs [28], was upregulated in irradiated HSPCs as we observed in our previous study (Fig 6B) [19]. Among the transcriptional regulators implicated in HSC self-renewal [29], [30], *MEIS1* was downregulated but *HOXA9* was not (Fig 6B).

## Discussion

Understanding the health effects of low levels of ionizing radiation remains an important issue. Although insufficient support of human HSPCs by the mouse BM niche cells is one of the limitations of xenotransplantation models, the humanized mouse model allows us to assess the long-term radiation effects as we previously reported [19].

In this study, non-lethal radiation doses of 0.5 and 1.0 Gy were shown to profoundly compromise the function of human HSPCs residing in the BM microenvironment. Human CD34^+^ HSPCs from irradiated humanized mice showed severely impaired reconstitution capacity in the secondary recipient mice and showed myeloid-biased repopulation at the expense of B-cell differentiation. Since these phenotypes are commonly observed in aged HSPCs, non-lethal ionizing irradiation could be one of the critical external stresses that promote aging of human HSPCs in the bone marrow niche.

Age-related decreases in regenerative capacity of HSCs have been, at least partly, attributed to accumulation of DNA damage [15], [16] or telomere shortening [31]. DNA damage triggers signaling cascades that activate tumor suppressor genes such as *p53* and *p16* ^*INK4A*^. Although its role in HSC aging is controversial [32], *p16* ^*Ink4a*^ expression increases with aging in HSCs and absence of p16^Ink4a^ ameliorates some of the age-related phenotypes of HSCs in mice. We have previously demonstrated that DNA damage accumulated in HSCs upon non-lethal doses of irradiation in humanized mice and that expression of *p16* ^*INK4A*^, a hallmark of aging of HSCs, was upregulated in HSPCs in irradiated mice [19]. In this study, *p16* ^*INK4A*^ was again significantly upregulated in CD34^+^ HSPCs which were exposed to 0.5 Gy irradiation in the BM niche of humanized mice; and this *p16* ^*INK4A*^ upregulation may account for the impaired HSC function. HOXA9 and MEIS1 are transcription factors essential for the self-renewal of HSCs [29], [30]. Of interest, while the expression of *HOXA9* was not altered, that of *MEIS1* was significantly downregulated in CD34^+^ HSPCs irradiated with 0.5 Gy, at both early and late time points post-irradiation (2 weeks post-irradiation and 3 months post-secondary transplantation, respectively). The transcription factor Meis1, belonging to the TALE family of non-Hox homeobox genes, protects and preserves HSCs by restricting oxidative metabolism [30]. These findings suggest that non-lethal doses of radiation could compromise, at least partly, transcriptional activation of genes crucial for the self-renewal capacity of HSCs.

Recently, HSCs have been demonstrated to be more heterogeneous in their capacity to repopulate hematopoiesis and produce a variety of progenies [33–36]. Notably, there are higher numbers of myeloid-biased HSCs in the aged BM compared with the young BM [24], [37], [38]. As a consequence, granulocyte/macrophage progenitors increase while common lymphoid progenitors decrease in aged mice. Therefore, aged HSCs show a greater propensity for myeloid differentiation compared with younger HSCs [7], [24], [25]. In our humanized mouse model, non-lethal radiation profoundly impaired the capacity of CD34^+^ HSPCs to give rise to B cells both *in vitro* and *in vivo*. These recent findings suggest that radiation promotes clonal expansion of myeloid-biased HSCs at the expense of lymphoid-biased HSCs via inducing DNA damage. Key regulators of B cell differentiation, *EBF1*, *PAX5* and *IL-7R* were all downregulated in CD34^+^ HSPCs upon 0.5 Gy of irradiation as was reported in human aged HSCs [25]. Downregulation of these B cell regulators could reflect the clonal expansion of myeloid-biased HSPCs in CD34^+^ HSPCs. Alternatively, radiation may alter transcriptional profiles of HSPCs crucial for their commitment and differentiation probably by inducing epigenetic changes at developmental regulator gene loci such as *EBF1* and *PAX5*.

In this study, we clearly demonstrated that non-lethal doses of radiation caused profound functional changes in HSCs in humanized mice. However, irradiation of humanized mice affects not only human HSPCs but also mouse BM niche and hematopoietic cells. Damaged mouse cells, particularly niche cells, could indirectly affect the human HSPCs. This possibility needs to be experimentally addressed in the future. To this end, NOD/Rag2^*null*^ IL2rγ^*null*^ mice, which are more resistant to radiation compared with NOG mice, would be useful. Finally, continuous efforts are required to clarify the effects of much lower doses of irradiation on HSCs. Such efforts would be valuable in order to prevent and contend with health hazards caused by occupational or unexpected radiation exposures.
